# The contribution of nano-zinc to alleviate salinity stress on cotton plants

**DOI:** 10.1098/rsos.171809

**Published:** 2018-08-08

**Authors:** M. M. Hussein, N. H. Abou-Baker

**Affiliations:** 1 Water Relations and Field Irrigation Department, National Research Centre, Dokki, Giza, Egypt; 2 Soils and Water Use Dept., Agric. Div., National Research Centre, Dokki, Giza, Egypt

**Keywords:** cotton, salinity, nano-zinc, growth, mineral status

## Abstract

To investigate the effect of nano-zinc fertilizer on growth, yield and mineral status of cotton plants grown under salt stress, a pot experiment was set up in the greenhouse of the National Research Centre. The treatments were as follows: (I) diluted seawater: 10% (S1), 20% (S2) and tap water as a control (S0), (II) 100 ppm (NZn1), 200 ppm (NZn2) nano-zinc and distilled water as a control (NZn0). Irrigation with 10 and 20% seawater decreased dry weight (DW) of leaves by 11.53 and 43.22%, while decreases in bolls were 15.50 and 71.65%, respectively. Except for root DW and top/root ratio, the measured growth parameters were increased as nano-zinc concentration increased. As for the interaction between treatments, the highest DW of stem, leaves and bolls resulted from the addition of NZn2 under normal condition, followed by NZn2 x S1 and the next was NZn2 x S2. The foliar application of 200 ppm nano-Zn led to mitigating the adverse effect of salinity and confirmed that diluted seawater could be used in the irrigation of cotton plant. However, phosphorus fertilizer should be added with nano-Zn application to avoid P/Zn imbalance. Some elements’ status and their ratios were recorded.

## 1. Introduction

Ecosystem processes changed by the climate change by increasing both biotic and abiotic stress [1]. Increased salinity of agricultural land is expected to have destructive global effects, resulting in loss of up to half of arable lands by the middle of the twenty-first century [2]. The adverse effects of salinity have been attributed to the increase of sodium and chloride that are considered the most important ions which induced several disorders in physiological processes of different plants [3]. Salinity leads to plant death by ionic and osmotic stress, which causes nutrient imbalance, membrane damage and enzymatic inhibition [4]. Soil salinity has been a main concern to global agriculture throughout human history [5]. Soil salinity reduces water availability to plants, increases ion toxicity levels, reduces absorption of essential nutrients and reduces crop yields and qualities [6].

Globally, cotton is a vital salt-tolerant and cash crop that is used in the reclamation of salt-affected soils. The world area of cotton (*Gossypium barbadense* L.) harvested in 2014 was 34 747 265 ha with the world production of 79 069 252 tonnes of seeds. Egypt's harvested area of cotton in 2014 was 155.054 ha with the seed production of 525 000 tonnes [7].

Zinc is an essential micronutrient for plants and animals. Decreasing zinc availability resulted in the reduction in crop yields and qualities [8]. Zinc plays an important role in the formation of chlorophyll, protein, lipid, carbohydrate, a cofactor in enzymes (DNA and RNA polymerase) and hormones’ actions. Zinc uptake by plants is influenced by the level of phosphate and calcium in the soil. There is a significant inverse relationship between phytic acid and calcium, phosphorus and zinc in plant, whereas a high calcium uptake could increase the required zinc, and vice versa. Symptoms of zinc deficiency are chlorosis or mottled leaves and abnormal roots. In humans, Zn usually is taken to stimulate the immune system [9,10].

Excessive application of chemical fertilizers is harmful to human health, animals, plants and the surrounding environment. Nano-fertilizer addition could be a promising way to solve these problems. Application of nano-fertilizers is one of the suitable methods for increasing resource use efficiency, plant production and reducing environmental pollution [11]. Powerful and inexpensive nano-fertilizers could replace traditional fertilizers and present a solution to increasing the huge amount of agricultural products added to soil [12]. Greatest growth and grain yield of wheat were produced by iron sulfate (8 gl^−1^) followed by nano-iron (2 gl^−1^), while the highest grain content of protein and iron was obtained by the nano-iron application [13].

Therefore, in this work, the authors report a novel approach in using nano-fertilizers under abiotic stress. The study also provides an estimation of the response of cotton growth, yield and mineral status of cotton leaves to nano-zinc application under salinity condition.

## 2. Material and methods

During the 2014 summer season, a greenhouse experiment was conducted at the National Research Centre to investigate the effect of nano-zinc fertilizer on cotton growth, yield and mineral status of plants leaves that are grown under salt stress. The treatments were as follows: (a) Salinity treatments: 10 and 20% of seawater (EC = 52 dSm^−1^ and pH = 8.37) and tap water (EC = 0.4 dSm^−1^ and pH = 7.9) as a control. (b) Fertilization treatments: two concentrations of nano-Zn were sprayed (100 and 200 ppm) after three weeks of sowing, and the second application was two weeks after the first application. The control plants were sprayed with the same quantity of distilled water.

Seeds of cotton (*Gossipyum barbadense L*.) were sown on 1 April. The pots (40 cm in diameter and 50 cm in height) were filled with 35 kg clay loam soil. The selected soil was taken from Kerdasa, Giza Governorate. The soil sample was air-dried, crushed and sieved to pass through a 2 mm sieve. This soil is characterized by the pH value equal to 7.88, EC 0.8 dSm^−1^, the soluble cation values were 2.6, 0.6, 3.3 and 1.5 meq l^−1^ soil for Na^+^, K^+^, Ca^2+^ and Mg^2+^, respectively, 0.7, 5.0 and 2.3 meq l^−1^ for HCO^3−^, Cl^−^ and SO_4_^2−^, respectively [14]. After germination, plants were thinned twice to be two plants per pot. Calcium superphosphate (15.5% P_2_O_5_) and potassium sulfate (48.5% K_2_O) in the rate of 1 g and 2 g, respectively, was broadcasted before sowing. Ammonium sulfate (20.6%N) was added in two equal portions, the first was three weeks after sowing and the second was two weeks later.

The dry weights of cotton root, stem and bolls were recorded. Two plants from every replicate were picked, cleaned and dried in an electric oven at 70°C until the weight became stable. Samples were ground in a stainless steel mill. A portion of the dried leaves was wet-digested with di-acid mixtures (sulfuric and perchloric acids), the digested aliquot was analysed for N, P, K, Ca, Na and Zn. Nitrogen was determined by micro-Kjeldahl apparatus, P was determined by the ascorbic acid method, K, Ca and Na elements were measured by flame-photometer and Zn was determined using atomic absorption spectrophotometer apparatus Perkin Elmer, Model AAS [15]. Concentrations and contents of the studied elements in cotton leaves at harvesting were calculated.

The experiment included nine treatments which were the combination of three treatments of salt stress and three treatments of nano-fertilizer. The experimental design was two-way randomized blocks in six replicates. The collected data were subjected to statistical analysis as described by Snedecor & Cochran [16].

## 3. Results and discussion

### 3.1. Effect of salt stress

A negative relationship was observed between salinity and dry weight (DW) of plant parts. The differences in leaves and bolls DW were significant. The solution with 10 and 20% seawater decreased DW of leaves by 11.53 and 43.22%, while decreases in bolls were 15.50 and 71.65%, respectively (table 1). Moreover, top and whole plant DW and top/root (T/R) ratio showed approximately the same trends in comparison with fresh water.

**Table 1.** Dry weight as affected by irrigation with diluted seawater.

	dry weight (g/plant)							
	root	stem	leaves	bolls	S + L	top	whole	T/R
diluted seawater	R	S	L	B		S + L+B	S + L+B + R	ratio
S0	2.17	11.42	5.46	29.03	16.88	45.91	48.08	21.16
S1	3.53	10.46	4.83	24.53	15.29	39.92	43.45	11.28
S2	2.54	5.97	3.10	8.23	9.07	7.30	19.84	6.81
LSD_5%_	n.s.	**2.34	**0.3	**1.62	—	—	—	—

**p ≤ 0.01.

It is clear from table 2 that N, K, Ca and Zn decreased with the irrigation using saline solutions, while P concentration increased slightly with moderate saline rate S1 without a significant difference to S0 and tended to decrease with the highest salinity treatment. On the opposite side, the Na concentration increased with salt treatments.

**Table 2.** Mineral concentrations in cotton as affected by irrigation with diluted seawater.

diluted seawater	N	P	K (%)	Ca	Na	Zn ppm
S0	3.21	0.22	3.29	1.93	0.52	37.6
S1	2.69	0.24	3.22	1.45	0.57	28.6
S2	2.66	0.15	3.06	1.56	0.58	31.9
LSD_0.05_	*0.4	***0.03	n.s.	**0.3	n.s.	**4.9

n.s.*p* > 0.05, *p ≤ 0.05, **p ≤ 0.01, ***p ≤ 0.001.

**Figure 1.**
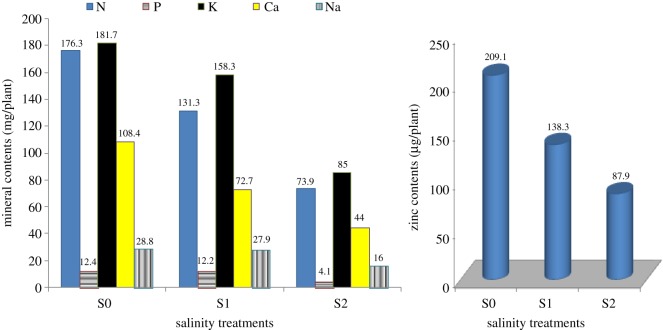
Mineral contents in cotton leaves irrigated with diluted seawater.

The content of all measured elements in plant leaves (mg/plant) follows the same trend as that of the dry weight of leaves, where they decreased significantly with increasing the salinity levels (figure 1).

Data presented in table 3 reveal that Na/K increased as the salinity level increased, but Na/Ca and K/Ca ratios increased with the moderate salinity level and tended to decrease, while it is still more than the control. However, Ca/(K + Na) decreased with the first level of salinity and tended to increase with the high level of salinity, but the values of this ratio are still less than those of the control. But, P/Zn ratio increased with the first level of salt and decreased by the high salt stress to be less than the control values**.**

**Table 3.** The ratios of some elements as affected by irrigation with diluted seawater.

diluted seawater	Na/K	Na/Ca	K/Ca	Ca/(K + Na)	P/Zn
S0	0.16	0.27	1.75	0.50	60.57
S1	0.18	0.41	2.29	0.38	84.93
S2	0.19	0.38	2.02	0.43	48.11
LSD_0.05_	n.s.	***0.1	**0.3	***0.1	***9.9

n.s.*p* > 0.05, ***p ≤ 0.001.

Salinity led to declining growth parameters, i.e. dry matter, uptake of N, P, K, Na and Ca, and yield of cotton as reported by Hussein *et al*. [17], who added that Na/K ratio increased as salt concentration increased in contrast to Ca/(Na + K) ratio that showed the opposite response. Also, salt stress results in a depression in plant height, root length, branch number, fresh and DW of shoots and roots of moringa [18,19]. The reduction in plant growth is a consequence of several physiological processes; photosynthetic activity, ion imbalance, water status, mineral nutrition, stomatal aperture and carbon allocation and use [20].

### 3.2. Effect of nano-zinc fertilizer

The measured growth parameters increased with increasing zinc concentration in the form of nano, except for root dry weight, or T/R ratio (table 4). The highest DW of root was obtained by spraying 100 ppm nano-zinc oxide. T/R ratio decreased with the first nano-zinc concentration and tended to increase markedly by using 200 ppm nano-zinc to be clearly more than the control. Pronounced increases in growth parameters, i.e. stem, leaves, bolls and whole plant DW, were shown with the increases in nano-fertilizer rates up to the highest level (NZn2) compared to plants receiving distilled water (NZn0).

**Table 4.** Growth responses to nano-zinc application.

	dry weight g/plant							
	root	stem	leaves	bolls	S + L	top	whole	T/R
nano-Zn treatments	R	S	L	B		S + L + B	S + L + B + R	ratio
NZn0	2.16	6.50	3.05	20.23	9.55	29.78	31.94	13.79
NZn1	3.70	9.18	4.90	23.13	14.08	37.21	40.91	10.06
NZn2	2.54	12.17	5.43	28.23	17.60	45.83	48.37	18.04
LSD_5%_	n.s.	**2.34	n.s.	***1.62	—	—	—	—

n.s.*p* > 0.05, **p ≤ 0.01, ***p ≤ 0.001.

Data in table 5 reported that N, K, Ca, Na and Zn concentrations augmented with nano-Zn treatments, while P% slightly increased with NZn1 and sharply decreased with NZn2. This may be due to the antagonistic effect between P and Zn.

**Table 5.** Element concentrations in cotton leaves as affected by nano-zinc application.

nano-Zn treatments	N	P	K %	Ca	Na	Zn ppm
NZn0	2.78	0.20	3.07	1.37	0.53	32.0
NZn1	2.89	0.23	3.24	1.73	0.55	32.8
NZn2	2.89	0.19	3.26	1.83	0.59	33.3
LSD_0.05_	n.s.	*0.03	n.s.	**0.3	n.s.	n.s.

n.s.*p* > 0.05, *p ≤ 0.05, **p ≤ 0.01.

**Figure 2.**
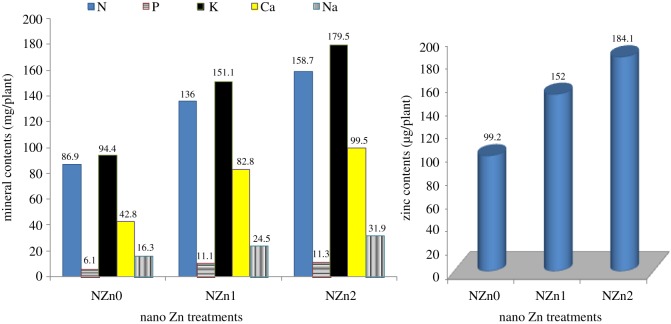
Element contents in cotton leaves as affected by nano-zinc application.

The content of leaves of all studied elements (mg/plant) follows the same trend of dry weight of leaves, where they increased significantly with the addition of nano-zinc compared to control and with increasing the foliar solution's concentration from 100 to 200 ppm (figure 2). The increasing percentages of adding NZn1 and NZn2 were 56.5 and 82.6% for N, 82.0 and 85.2% for P, 60.1 and 90.1% for K, 93.5 and 132.5% for Ca, 50.3 and 95.7% for Na and 53.2 and 85.6% for Zn. Interestingly, calcium is the most influenced nutrient with nano-Zn application.

Examination of data in table 6 shows that Na/Ca and K/Ca ratios decreased with Zn nano-spraying without significant differences between its application rates, but the opposite was true for Ca/(K + Na) ratio. Zinc application did not affect Na/K ratio; meanwhile, P/Zn ratio was increased by the NZn1 and decreased with the highest level of nano-Zn. Also, its value became less than that of the control.

**Table 6.** The ratios of some elements as affected by nano-zinc application.

nano-Zn treatments	Na/K	Na/Ca	K/Ca	Ca/(K + Na)	P/Zn
NZn0	0.17	0.40	2.29	0.38	61.38
NZn1	0.17	0.32	1.91	0.46	72.03
NZn2	0.18	0.34	1.87	0.48	60.20
LSD_0.05_	n.s.	*0.1	*0.3	**0.1	*9.9

n.s.*p* > 0.05, *p ≤ 0.05, **p ≤ 0.01.

Growth parameters of savory plant; height, leaf number, leaves fresh and DW, chlorophyll, essential oil and phosphorus content were improved by nano-zinc application [21]. The height, fresh and dry weights of treated cotton plants increased in control < mineral Zn < Zn-chelate < nano-Zn chelate in that order [22]. Foliar Zn application increased Zn concentration and protein, carbohydrate metabolism, but decreased P% in grains of wheat compared with Zn alone [23]. The positive response of nano-ZnO compared to ZnSO_4_ and ZnO of normal size on chickpea may be attributed to low ROS (reactive oxygen species) levels, which led to lower lipid peroxidation, MDA (malondialdehyde), activity of prominent antioxidant enzymes and superoxide dismutase as discussed by Burman *et al*. [24]. Also, zinc addition affects auxin (growth regulator) biosynthesis positively; this can promote mineral absorption, cell division and thus enhance plant growth [25,26]. It also enables the plant to maintain the plasma membrane integrity [27]. In tomato plants, inadequate Zn is correlated with a reduction in IAA content which tends to increase after zinc is resupplied [9]. In cotton, Rezaei & Abbasi [22] reported that application of nano-chelate zinc improves physiological processes in cotton plant; increases chlorophyll content and antioxidant activity of catalase, peroxidase and polyphenol oxidase.

### 3.3. The interaction effect between the examined parameters

As for the interaction effect between nano-zinc application under two levels of salinity when compared with irrigation with fresh water as a control, it could be stated that the addition of nano-zinc is more effective in the whole plant dry mass under 20% diluted seawater treatment than that of 10% diluted seawater treatment or freshwater irrigation (table 7). In spite of the insignificant differences, DW of different cotton plants as well as total dry mass was improved by the application of nano-fertilizer under different salinity levels. The highest values of all the measured growth parameters were produced by the interaction S0 × NZn2 except for root and stem dry weight that produced their highest values by the second interaction S1 × NZn1 and S1 × NZn2.

**Table 7.** Growth responses to nano-zinc application and irrigation with diluted seawater.

		dry weight g/plant							
		root	stem	leaves	bolls	S + L	top	whole	T/R
diluted seawater	nano-Zn treatments	R	S	L	B		S + L + B	S + L+B + R	ratio
S0	NZn0	2.33	7.83	3.87	24.6	11.70	40.10	42.43	14.32
	NZn1	2.80	11.87	5.87	28.4	17.74	51.84	58.20	18.51
	NZn2	1.90	14.57	6.73	34.1	21.30	55.40	57.30	29.16
S1	NZn0	2.27	7.67	2.76	22.3	10.43	32.73	35.00	14.42
	NZn1	4.07	11.73	5.60	24.2	17.31	41.53	45.60	10.20
	NZn2	2.83	12.97	6.20	27.1	19.17	46.27	49.10	16.34
S2	NZn0	1.87	4.00	2.53	14.4	6.53	20.93	22.80	11.19
	NZn1	3.63	3.93	2.37	16.8	6.30	23.10	26.73	6.36
	NZn2	2.90	8.97	3.37	23.5	12.34	35.84	38.74	12.36
LSD_5%_		n.s.	n.s.	n.s.	n.s.	—	—	—	—

n.s.*p* > 0.05.

The interaction effect of nano-fertilizers and salinity on the mineral concentration of cotton plants is illustrated in table 8. All mineral concentrations were not affected significantly by nano-zinc treatments under applied salinity levels except for P that produced its highest value by the application of NZn1 with the medium salinity degree. Nitrogen percentage decreased similarly with both salt concentrations, so its highest value resulted from irrigation with tap water plus application of NZn1. Addition of the high level of nano-zinc (NZn2) without salinity stress (S0) enhanced the other values of determined element concentrations.

**Table 8.** Element concentrations in cotton leaves irrigated with diluted seawater as affected by nano-zinc application.

diluted seawater	nano-Zn treatments	N	P	K %	Ca	Na	Zn ppm
S0	NZn0	3.17	0.22	3.15	1.62	0.51	34.9
	NZn1	3.30	0.22	3.26	2.00	0.45	37.3
	NZn2	3.16	0.23	3.45	2.16	0.60	40.6
S1	NZn0	2.66	0.21	3.05	1.20	0.55	29.3
	NZn1	2.68	0.29	3.34	1.70	0.59	28.3
	NZn2	2.73	0.23	3.26	1.45	0.57	28.2
S2	NZn0	2.52	0.16	3.00	1.28	0.54	31.8
	NZn1	2.69	0.18	3.11	1.50	0.60	32.7
	NZn2	2.77	0.12	3.08	1.89	0.59	31.0
LSD_0.05_	****	n.s.	*0.05	n.s.	n.s.	n.s.	n.s.

n.s.*p* > 0.05, *p ≤ 0.05.

**Figure 3.**
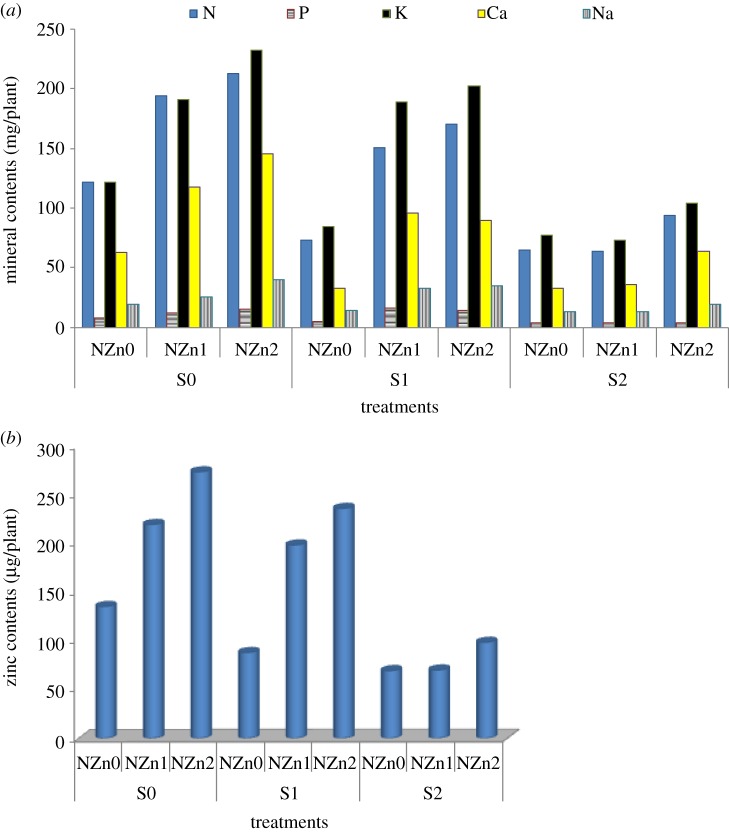
(*a*) Element contents in cotton leaves irrigated with diluted seawater as affected by nano-zinc application. (*b*) Zinc contents in cotton leaves irrigated with diluted seawater as affected by nano-zinc application.

Figure 3*a*,*b* illustrates the interaction effect of nano-fertilization and salinity on the mineral content of cotton leaves. This interaction significantly affected the content of minerals in leaves except for nitrogen. It is clear that salt stress decreased the content of minerals and vice versa for nano-zinc treatment. The lowest values of N, P and K content were produced by S2 × NZn1, while the lowest values of Ca, Na and Zn contents were produced by S2 × NZn0.

The interaction effect was not significant except for the P/Zn ratio, which decreased by Zn treatment. Furthermore, this ratio was increased by the first Zn treatment and decreased with the second one under either moderate salinity or the highest rate (table 9). In another study on cotton, addition of nano-Zn, nano-Si and a combination of them had no significant effect on any measured parameters [28].

**Table 9.** The ratios of some elements as affected by nano-zinc application and irrigation with diluted seawater.

diluted seawater	nano-Zn treatments	Na/K	Na/Ca	K/Ca	Ca/(K + Na)	P/Zn
S0	NZn0	0.16	0.31	1.95	0.44	64.07
	NZn1	0.14	0.22	1.69	0.54	58.37
	NZn2	0.17	0.28	1.61	0.53	59.27
S1	NZn0	0.18	0.46	2.55	0.33	70.03
	NZn1	0.18	0.35	1.96	0.43	103.30
	NZn2	0.18	0.42	2.36	0.38	81.47
S2	NZn0	0.18	0.43	2.36	0.36	50.03
	NZn1	0.19	0.40	2.07	0.41	54.43
	NZn2	0.19	0.31	1.64	0.52	39.87
LSD_0.05_		n.s.	n.s.	n.s.	n.s.	*17.2

n.s.*p* > 0.05, *p ≤ 0.05.

Increasing density and reactivity of the specific surfaces of nanoparticles led to enhanced plant physiology and performance, thus increasing its ability to mitigate salinity. Moringa accumulates lower concentration of Na^+^ and Cl^−^ and higher amount of N, P, K, Ca, Mg, Fe and Zn upon foliar application of nano-ZnO to Hogland solution compared with those receiving Hogland solution only, under salt stress condition [19]. Arough *et al*. [29] reported that under high salinity level (5.55 dS m^−1^), biofertilizer and 0.8 g l^−1^ nano-zinc oxide increased grain yield of Triticale (a hybrid of wheat and rye) by 39% compared with the control (without any additions). Application of nano-zinc at rates of 25 or 50 mg l^−1^ caused significant changes in fresh and dry weight as well as in relative water content of rice [30] in biomass production of sunflower [31], in grain yield of wheat under salinity stress [32] and in yield of maize under drought [33]. These enhancements in growth, yield and quality of different plant kinds with nano-fertilizer addition may be due to (1) increasing the nutrient use efficiency [34,35], (2) minimizing soil toxicity induced by overdosage of fertilizer addition [34], (3) improving levels of antioxidant enzyme activities, consequently protecting plants from damage caused by reactive oxygen species [30].

## 4. Conclusion

The foliar application of nano-Zn led to mitigate the adverse effect of salinity and confirmed that diluted seawater could be used in the irrigation of the cotton plant. Also, nano-Zn enhanced cotton growth parameters and yield under stress condition. However, increasing the application rate of nano-Zn may reduce P absorption and translocation to leaves and consequently reduce the P/Zn ratio. It should be mentioned that an additional dose of phosphorus fertilizer with nano-Zn could be used to avoid the P/Zn imbalance. Further studies on the effect of nano-Zn on health and the environment of users are required.

## Data accessibility

All data are in the tables and figures in the main text.

## References

[1] Robroek BJ, Jassey VE, Beltman B, Hefting MM 2017 Diverse fen plant communities enhance carbon-related multifunctionality, but do not mitigate negative effects of drought. R. Soc. open sci. 4,170449 (10.1098/rsos.170449) 29134063 PMC5666246

[2] Mahajan S, Tuteja N 2005 Cold, salinity and drought stresses: an overview. Arch. Biochem. Biophys. 444, 139–158. (10.1016/j.abb.2005.10.018) 16309626

[3] Tavakkoli E, Rengasamy P, McDonald GK 2010 High concentrations of Na^+^ and Cl^–^ ions in soil solution have simultaneous detrimental effects on growth of faba bean under salinity stress. J. Exp. Bot. 61, 4449–4459. (10.1093/jxb/erq251) 20713463 PMC2955754

[4] Hasanuzzaman M, Hossain MA, Fujita M 2012 Exogenous selenium pretreatment protects rapeseed seedlings from cadmium-induced oxidative stress by up regulating the antioxidant defense and methylglyoxal detoxification systems. Biol. Trace Elem. Res. 149, 248–261. (10.1007/s12011-012-9419-4) 22535598

[5] Hasanuzzaman M, Nahar K, Fujita M 2013 Plant response to salt stress and role of exogenous protectants to mitigate salt-induced damages. In Ecophysiology and responses of plants under salt stress (eds Ahmad P, Azooz M, Prasad M), pp. 25–87. New York, NY: Springer.

[6] Grattan SR, Grieve CM 1999 Mineral nutrient acquisition and response by plant grown in saline environments. Agric. Ecosys. Environ. 38, 275–300. (10.1016/0167-8809(92)90151-Z)

[7] FAO. 2017 Production/yield quantities of cotton in world. http://www.fao.org/faostat

[8] Sarwar M 2011 Effects of zinc fertilizer application on the incidence of rice stem borers (*Scirpophaga* species)(Lepidoptera: Pyralidae) in rice (*Oryza sativa* L. crop). J. Cereals and Oilseeds 2, 61–65.

[9] Marschner H 1995 Mineral nutrition of higher plant, 2nd edn. London, UK: Acadamic Press.

[10] Soetan KO, Olaiya CO, Oyewole OE 2010 The importance of mineral elements for humans, domestic animals and plants: a review. Afr. J. Food Sci. 4, 200–222.

[11] Taha RA, Hassan MM, Ibrahim EA, Abou-Baker NH, Shaaban EA 2016 Carbon nanotubes impact on date palm *in vitro* cultures. Plant Cell, Tissue and Organ Culture (PCTOC) 127, 525–534. (10.1007/s11240-016-107703)

[12] El-Kereti MA, El-Feky SA, Khater MS, Osman YA, El-Sherbini ESA 2013 ZnO nanofertilizer and He Ne laser irradiation for promoting growth and yield of sweet basil plant. Recent Pat. Food Nutr. Agric. 5, 169–181. (10.2174/2212798405666131112142517) 24215471

[13] Ghafari H, Razmjoo J 2013 Effect of foliar application of nano-iron oxidase, iron chelate and iron sulphate rates on yield and quality of wheat. Intr. J. Agron. Plant Prod. 4, 2997–3003.

[14] Page AL, Miller RH, Keeny DR 1982 Methods of soil analysis, part II chemical and microbiological properties, 2nd edn Madison, Wisconsin: Amer. Soc. Agron Monograph No. 9.

[15] Cottenie A, Verloo M, Kiekens L, Velghe G, Camerlynck R 1982 Chemical analysis of plants and soils. Ghent, Belgium: Laboratory of Analytical and Agrochemistry State University.

[16] Snedecor GW, Cochran WG 1980 Statistical methods, 6th edn Amess, IA: lowa State University.

[17] Hussein MM, Mehanna H, Abou-Baker NH 2012 Growth, photosynthetic pigments and mineral status of cotton plants as affected by salicylic acid and salt stress. J. Appl. Sci. Res. 8, 5476–5484.

[18] Hussein MM, Abou-Baker NH 2014 Growth and mineral status of moringa plants as affected by silicate and salicylic acid under salt stress. Int. J. Plant Soil Sci. 3, 163–177. (10.9734/IJPSS/2014/6105)

[19] Soliman AS, El-Fiky S, Dariesh E 2015 Alleviation salt stress in *Moringa pergrina* using foliar application of nano-fertilizer. J. Hort. Forest. 7, 36–47. (10.5897/JHF2014.0379)

[20] Bregnoli E, Loutari M 1991 Effect of salinity on stomatal conductance, photosynthetic capacity and carbon isotope discrimination of salt tolerant (*Gossypium hirsotum* L.) and salt sensitive (*Phaseolus vulgaris* L.) C3 non-halophytes. Plant Physiol. 95, 628–635. (10.1104/pp.95.2.628) 16668029 PMC1077578

[21] Vafa ZN, Sirousmehr AR, Ghanbari A, Khammari E, Falahi N 2015 Effect of nano-zinc and humic acid in quantitative and qualitative characteristics of savory (*Satureja hortensis* L.) Int. J. BioSci. 6, 124–136.

[22] Rezaei M, Abbasi H 2014 Foliar application of nanochelate and non-nanochelate of zinc on plant resistance physiological processes in cotton (*Gossipium hirsutum* L.). Iran. J. Plant Physiol. 4, 1137–1144.

[23] Li M, Wang S, Tian X, Zhao J, Li H, Guo C, Chen Y, Zhao A 2015 Zn distribution and bioavailability in whole grain and grain fractions of winter wheat as affected by applications of soil N and foliar Zn combined with N or P. J. Cereal Sci. 61, 26–32. (10.1016/j.jcs.2014.09.009)

[24] Burman U, Saini M, Kumar P 2013 Effect of zinc oxide nanoparticles on growth and antioxidant system of chickpea seedlings. Toxicol. Environ. Chem. 95, 605–612.

[25] Cakmak I 2008 Enrichment of cereal grains with zinc: agronomic or genetic bio-fortification. Plant Soil 302, 1–17. (10.1007/s11104-007-9466-3)

[26] El-Tohamy WA, El-Greadly NHM 2007 Physiological responses, growth, yield and quality of snap bean in response to foliar application of yeast, vitamin E and zinc under sandy soil conditions. Aust. J. Basic Appl. Sci. 1, 249–299.

[27] Raliya R, Tarafdar JC 2013 ZnO nanoparticle biosynthesis and its effect on phosphorus- mobilizing enzyme secretion and gum contents in clusterbean (*Cyamopsis tetragonoloba* L). Agric. Res. 20, 48–57. (10.1007/s40003-012-0049-z)

[28] Siskani A, Seghatoleslami M, Moosavi G 2015 Effect of deficit irrigation and nano fertilizers on yield and some morphological traits of cotton. Biol. Forum Res. Trend 7, 1710–1715.

[29] Arough YK, Sharifi RS, Sedghi M, Barmaki M 2016 Effect of zinc and bio fertilizers on antioxidant enzymes activity, chlorophyll content, soluble sugars and proline in *triticale* under salinity condition. Notulae Botanicae Horti Agrobotanici Cluj-Napoca 44, 116 (doi:10.15835/nbha44110224)

[30] Upadhyaya H, Shome S, Tewari S, Bhattacharya MK, Panda SK 2015 Effect of Zn nano-particles on growth responses of rice. In Nanotechnology: Novel Perspectives and Prospects (eds Singh B, Kaushik A, Mehta SK, Tripathi SK), pp. 508–512. New Delhi, India: McGraw Hill Education.

[31] Torabian S, Zahedi M, Khoshgoftarmanesh A 2016 Effect of foliar spray of zinc oxide on some antioxidant enzymes activity of sunflower under salt stress. J. Agric. Sci. Technol. 18, 1013–1025.

[32] Babaei K, Sharifi RS, Pirzad A, Khalilzadeh R 2017 Effects of bio fertilizer and nano Zn-Fe oxide on physiological traits, antioxidant enzymes activity and yield of wheat (*Triticum aestivum* L.) under salinity stress. J. Plant Interact. 12, 381–389. (10.1080/17429145.2017.1371798)

[33] Farnia A, Omidi MM, Farnia A 2015 Effect of nano-zinc chelate and nano-biofertilizer on yield and yield components of maize (*Zea Mays* L.) under water stress condition. Indian J. Nat. Sci 5, 4614–4646.

[34] Naderi MR, Danesh-Shahraki A 2013 Nanofertilizers and their roles in sustainable agriculture. Int. J. Agri. Crop Sci. 5, 2229.

[35] Huang S, Wang L, Liu L, Hou Y, Li L 2015 Nanotechnology in agriculture, livestock, and aquaculture in China: a review. Agron. Sustainable Dev. 35, 369–400. (10.1007/s13593-014-0274-x)

